# Clinical applications of palifermin: amelioration of oral mucositis and other potential indications

**DOI:** 10.1111/jcmm.12169

**Published:** 2013-11-19

**Authors:** Saroj Vadhan-Raj, Jenna D Goldberg, Miguel-Angel Perales, Dietmar P Berger, Marcel RM Brink

**Affiliations:** a Department of Sarcoma Medical Oncology, Section of Cytokines and Supportive Oncology, Division of Cancer Medicine, The University of Texas MD Anderson Cancer CenterHouston, TX, USA; b Department of Medicine, Adult Bone Marrow Transplantation Service, Memorial Sloan-Kettering Cancer CenterNew York, NY, USA; c Weill Cornell Medical CollegeNew York, NY, USA; d Amgen Inc.Thousand Oaks, CA, USA; e Department of Oncology, Genentech Inc.South San Francisco, CA, USA

**Keywords:** palifermin, KGF, oral mucositis, dysphagia, GVHD, HSCT, immune reconstitution, mucositis, palliative care

## Abstract

Mucositis is one of the most significant toxicities in cancer patients undergoing cytotoxic treatment. It can have a negative impact on both quality of life and health economics. Severe oral mucositis can contribute to hospitalization, need for narcotic analgesics, total parentral nutrition, suboptimal delivery of anti-neoplastic treatment, and morbidity and mortality. Palifermin, a recombinant derivative of human keratinocyte growth factor, is the first active agent approved by the FDA for the prevention of severe oral mucositis in patients undergoing haematopoietic stem cell transplantation (HSCT). Several studies have also shown significant reduction in the incidence, severity and/or duration of oral mucositis in other high-risk settings such as concurrent chemoradiotherapy (CT/RT) for patients with head and neck cancer, and use of mucotoxic chemotherapeutic agents such as doxorubicin in sarcoma and fluorouracil for the treatment of colorectal cancer. The reduction in mucositis has translated into amelioration of symptoms and improvement in daily functioning as measured by patient-reported outcome in multiple studies. The clinical response to palifermin appears to be related in part to epithelial proliferation and mucosal thickening. Palifermin also has other potential clinical applications including the acceleration of immune reconstitution and inhibition of graft-versus-host disease in patients undergoing HSCT, and mitigation of dysphagia in lung cancer patients treated with concurrent CT/RT. Palifermin is generally well tolerated with mild-to-moderate skin and oral adverse events. Future studies may expand the use of palifermin into other areas that would benefit from its cytoprotective and regenerative effects.

## Introduction

Mucositis is one of the most significant non-haematological toxicities in cancer patients, resulting from epithelial injury caused by cytotoxic chemotherapy (CT) and/or radiation therapy (RT). It can have profound clinical and economic implications 1,2. The severity of mucositis ranges from mild erythema and/or soreness to diffuse erythema and ulcerations that can be very painful and debilitating. Severe oral mucositis is associated with significant morbidities, including reduced oral intake and increases in the use of narcotic pain medications, risk of infections and hospitalization leading to a negative impact on quality of life 4,5. Severe mucositis can also result in CT dose reduction and treatment delays, potentially leading to poor treatment outcome. Mucositis is a significant driver of the health care cost by increasing emergency room visits, hospitalization, the use of antibiotics and parental nutrition 4–7.

Although the precise number of cancer patients receiving CT and/or RT in the United States annually is unknown, based on analysis by the Medical Panel Expenditures Survey 8, each year over 1.1 million patients are estimated to receive CT or RT for cancer. The incidence and severity of mucositis varies depending on the type and intensity of cancer therapy, in addition to host and disease-related factors. Oral mucositis is almost universal in patients undergoing an intensive conditioning regimen consisting of high-dose CT with or without RT prior to haematopoietic stem cell transplantation. Total body irradiation (TBI)-based preparative regimens are associated with rates of oral mucositis as high as 98% and over 50% have a severe grade 4–9. The incidence of mucositis is also very high (80–100%) in patients with head and neck cancer receiving concurrent CT/RT 6,10. In addition, patients receiving certain myelotoxic, multi-cycle treatment regimens for sarcoma, lymphoma, breast cancer or colorectal cancer are also at increased risk for mucositis. Despite its high incidence and significant impact on clinical morbidities and health economics, there have not been standard, government-approved, effective therapies to control this debilitating side effect in the majority of cancer patients undergoing anti-neoplastic treatment 12–13.

Clinical development of local therapies with topical agents to alleviate mucositis has met with limited success, providing only temporary relief of the problem 12. Among the strategies that have been tried for prevention of mucositis include cryotherapy (use of ice chips), low level lesser therapy (LLLT), Gelclair (bioadherent gel), glutamine (amino acid rich in nitrogen), AES-14 (L-glutamine combined with a vehicle, Saforis) and benzydamine (non-steroidal anti-inflammatory drug). In addition, considering the close relationship between neutrophil recovery and resolution of mucositis, myeloid growth factors, G-CSF and GM-CSF have been investigated and suggested to have some beneficial effect on mucositis 14. However, none of these strategies has been uniformly successful and the treatment of mucositis has remained an unmet need.

In the last decade, progress has been made in understanding the pathobiology of mucositis and exploring the use of epithelial cell growth factors for the amelioration of this malady 1–3. Of these agents, palifermin, a truncated derivative of keratinocyte growth factor (KGF, also known as FGF7) with enhanced stability (Swedish Orphan Biovitrum product information), has shown the most promise. Keratinocyte growth factor is a 28-kD protein produced by mesenchymal cells that stimulates cellular responses *via* its receptor, FGFR2b 15,16, which is expressed almost exclusively by epithelial cells in a wide variety of tissues, including the buccal mucosa, oesophagus, stomach, intestine, salivary gland, lung, liver, pancreas, kidney, bladder, prostate, mammary gland, skin, lens of the eye and thymus 18–19. It is not expressed by haematopoietic cells or most other cells of mesenchymal origin 20. The exact mechanisms of action of KGF have not been fully elucidated, although it is known to promote cell proliferation and cytoprotection, inhibit apoptosis and modulate the cytokine profile 17–21. Endogenous KGF is up-regulated following injury and appears to play a key role in the healing process 17. A series of pre-clinical studies demonstrated that palifermin decreased the mucotoxic effects of various combinations of CT and/or RT 22–26, providing the foundation for several clinical trials designed to test its safety and efficacy in cancer patients.

In 2004, the U.S. Food and Drug Administration approved the use of palifermin to reduce the incidence and duration of severe oral mucositis in patients with haematological malignancies undergoing myeloablative therapy followed by haematopoietic stem cell support. Palifermin has also shown efficacy in ameliorating oral mucositis in patients receiving concurrent CT/RT or multi-cycle CT to treat a subset of solid tumours, although regulatory approval has not yet been granted for its use in these settings. In addition, pre-clinical research has led to the initiation of clinical trials to investigate its utility in promoting immune reconstitution following haematopoietic stem cell transplantation and reducing graft-versus-host disease (GVHD) after allogeneic transplantation. The main focus of this article is the clinical experience with palifermin in prevention and management of oral mucositis. Its potential application in other areas will also be briefly reviewed.

## Haematopoietic stem cell transplantation (HSCT)

### Oral mucositis following autologous transplantation

High-dose therapy followed by autologous stem cell transplantation (HDT-ASCT) is an established treatment for many haematological malignancies. Mucositis, which results from injury to epithelial cells that line the oral cavity and gastrointestinal (GI) tract, can be a complication of both high-dose CT and radiation-based conditioning for HDT-ASCT. Mucositis can cause patients to suffer from oral pain, significant mouth sores, nausea and anorexia 4,27. A loss of integrity of the GI tract could lead to increased infections through translocation of bacteria that line the gut into the systemic circulation. Together, these complications increase morbidity and prolong the hospital stay.

The ability of KGF to mitigate mucositis following CT- and RT-induced GI injury was investigated in pre-clinical murine models. In these studies, administration of KGF prior to GI injury significantly decreased weight loss after injury and increased weight gain during recovery 23. In addition, an increase in villus height and crypt depth was noted. Animals who received KGF had 60% increased survival compared with control animals.

These results led to clinical studies of palifermin. The maximally tolerated dose for palifermin was established in two separate phase I clinical trials 29–30. The first study randomized 81 patients with metastatic colorectal cancer treated with fluorouracil (5-FU) to escalating doses of palifermin (2:1 randomization of palifermin to placebo) and showed a trend towards a decreased frequency of ulcerative mucositis (WHO grade 2–4): 43% *versus* 67%, *P* = 0.06. The maximum tolerated dose was 80 mcg/kg/day for 3 days 29. The second trial was performed in patients receiving BEAM (carmustine, etoposide, cytarabine, melphalan) as high-dose therapy followed by an autologous peripheral blood stem cell transplant for lymphoma 30. Patients were randomized to receive either placebo or escalating doses of palifermin using two schedules. Patients received three daily doses of palifermin either preceding the preparative regimen or both before and after the preparative regimen, the latter set being administered immediately after transplant, starting on the day of stem cell infusion. Again, the maximum tolerated dose was determined to be 80 mcg/kg/day for 3 days. Additional patients were randomized to the 60 mcg/kg/day (*n* = 30) and 80 mcg/kg/day (*n* = 49) pre-BEAM cohorts for a preliminary analysis of efficacy. At 60 mcg/kg/day × 3 days, 13% of patients experienced ulcerative oral mucositis and the mean (±SE) duration was 0.8 ± 0.5 days for palifermin recipients compared with a 51% incidence and 4.6 ± 1.8 days duration in placebo recipients. Based on the safety profile and preliminary efficacy, a 60 mcg/kg/day dose was chosen for use in future studies.

Subsequently, a double-blind, placebo-controlled phase II study evaluated the efficacy of palifermin in reducing mucositis in 129 patients undergoing a TBI-based HDT-ASCT 31. Patients were randomized in a 1:1:1 ratio to palifermin at a 60 mcg/kg daily dose for 3 days prior to the preparative regimen, palifermin 60 mcg/kg/day for 3 days prior to the preparative regimen plus palifermin at 60 mcg/kg/day 3 days post stem cell infusion or placebo. Patients who received either schedule of palifermin had a significant reduction in the duration of WHO grade 3–4 mucositis compared with the patients who received placebo. For the patients who received pre- and post-palifermin, the difference was 4 days *versus* 7.7 days (*P* = 0.001). The patients who received pre-palifermin only had a mean 5 days of grade 3–4 mucositis compared with placebo (*P* = 0.04). In addition, pre/post-treatment with palifermin was associated with a decrease in the use of total parenteral nutrition (TPN; 7.7 days *versus* 11.3 days) and intravenous narcotic (8.3 days *versus* 12.1 days).

The clinical efficacy of palifermin was then demonstrated in a landmark randomized double-blind, placebo-controlled multicenter phase III trial performed in 212 patients who received a high-dose preparative regimen consisting of fractionated TBI (1200 cGy total dose), high-dose etoposide (60 mg/kg) and high-dose cyclophosphamide (100 mg/kg) followed by ASCT for the treatment of haematological malignancies (Table 1) 32. Patients were randomized to receive either palifermin (*n* = 106) or placebo (*n* = 106). Palifermin was administered as a daily injection of 60 mcg/kg for three consecutive days prior to the start of the preparative regimen and for three consecutive days following transplant starting on the day of stem cell infusion. In patients who received palifermin, there was a significant reduction in median days of WHO grade 3–4 oral mucositis (3 *verses* 9 days, *P* < 0.001), a lower incidence of WHO grade 3–4 oral mucositis (63% *versus* 98%, *P* < 0.001) and a lower incidence of WHO grade 4 oral mucositis (20% *versus* 62%, *P* < 0.001). In a separate publication, the authors reported that patients documented a statistically significant improvement in daily activities such as swallowing, drinking, eating, talking and sleeping (*P* < 0.001) 33. Following this trial, palifermin was approved by the U.S. Food and Drug Administration for the prevention of CT- and/or RT-induced mucositis following both HDT-ASCT and allo-HSCT. Also, a separate economic analysis based on this study demonstrated that the benefit of decreased mucositis may outweigh the additional cost of palifermin administration 34. A non-significant mean savings of $3595 per patient [95% confidence interval (CI): $2090–$5103] was observed. In Europe, palifermin was approved for autologous transplantation only.

**Table 1:** Palifermin phase II and III clinical trials: published randomized, double-blind, placebo-controlled studies

Disease	Treatment	Palifermin dose/Schedule	Endpoints/Observations*	Side effects	Reference
Autologous transplant (HDT-ASCT)					
Hematologic cancer (Phase III)	TBI (12 Gy) CT: etoposide: 60 mcg/kg × 1 day cyclophosphamide: 100 mg/kg × 1 day	60 mcg/kg/day × 3 days pre & × 3 days post transplant	Incidence, grade 3 or 4 OM: 63% *versus* 98%, *P* < 0.001 Incidence, grade 4 OM: 20% *versus* 62% Duration, severe OM (median): 3.0 days *versus* 9.0 days, *P < 0.001* TPN: 31% *versus* 55%, *P* < 0.001 Morphine equivalents (median): 212 mg *versus* 535 mg, *P < 0.001* Better PRO with palifermin	Skin: rash, pruritis erythema Mouth: taste alteration, white film, thick tongue Edema	32
Multiple myeloma (Phase III)	CT: High dose melphalan 200 mg/m^2^ × 1 day, if CC ≥ 30 ml/min.; 140 mg/m^2^ × 1 day, if CC < 30 ml/min.)	60 mcg/kg/day × 3 day pre only, or × 3 days pre & × 3 days post transplant	Incidence, grade 3 or 4 OM: 24% *versus* 38% *versus* 37%, *P* = NS Duration, Severe OM (mean): 1.9 days *versus* 2.7 days *versus* 2.4 days, *P* = NS Similar PRO in all groups	Skin, mouth and edema: as above	35
Solid tumors					
Head/Neck cancer(Phase II)	Standard RT: 2 Gy/day, to 70 Gy, or Hyperfrac RT: 1.25 Gy bid, to 72 Gy CT: cisplatin: week 1, 5 20 mg/m^2^ × 4 days 5-FU: week 1, 5: 1000 mg/m^2^ × 4 days	60 mcg/kg/week × 10 weeks	Incidence, grade 3 or 4 OM: 83% *versus* 84% (SRT) 40% *versus* 77% (HRT) Duration, severe OM: 3.4 weeks *versus* 3.6 weeks (SRT) <1 week *versus* 3.0 weeks (HRT) Duration, grade ≥ 2 OM: 10.0 weeks *versus* 8.3 weeks (SRT) 4.6 weeks *versus* 8.1 weeks (HRT) Trends suggest symptomatic benefit with HRT, not SRT	Similar in palifermin and placebo arms	70
Head/Neck cancer(Phase III)	RT: 2 Gy/day, to 66 Gy CT: cisplatin: day 1, 22(and 43 if incomplete resection), 100 mg/m^2^[CT/RT followed local complete or incomplete resection]	120 mcg/kg/week × 7 weeks	Incidence, grade 3 or 4 OM: 51% *versus* 67%, *P* = 0.027 Duration, severe OM (median): 4.5 days *versus* 22.0 days, *P* = 0.037 Similar PRO in both groups	Similar in palifermin and placebo arms	71
Head/Neck cancer(Phase III)	RT: 2 Gy/day, to 70 Gy CT: cisplatin: day 1, 22, 43:100 mg/m^2^	180 mcg/kg/week × 7 weeks	Incidence, grade 3 or 4 OM: 54% *versus* 69%, *P* = 0.041 Duration, severe OM (median): 5 days *versus* 26 days, *P* = 0.016 Similar PRO in both groups	Rash, flushing, taste disturbance	72
Non-small cell lung cancer(Phase II)	RT: 2 Gy/day, to 60–66 Gy CT: paclitaxel: 50 mg/m^2^ q week × 7 weeks carboplatin: AUC 2.0 q week × 7 weeks [Followed by 2 cycles of consolidation CT: paclitaxel: 225 mg/m^2^ carboplatin: AUC 6.0]	180 mcg/kg/week × 7 weeks	Incidence, grade ≥ 2 dysphagia: 61% *versus* 70%, *P* = 0.36 Incidence, grade ≥ 3 dysphagia: 22% *versus* 28%, *P *= 0.50 Duration, grade ≥ 2 dysphagia: 25.3 days *versus* 32.4 days, *P* = 0.32 Unplanned RT breaks: 18% *versus* 33%, *P *= 0.11 Received at least 6 CT doses: 71% *versus* 61%	Rash, erythema, flushing, diarrhea	80
Colorectal cancer (Phase II)	CT: two cycles, each 28 days 5-FU: 425 mg/m^2^ × 5 days LV: 20 mg/m^2^ × 5 days (each given on cycle days 1–5)	40 mcg/kg/day × 3 days pre-CT	Incidence, grade ≥ 2 OM: Cycle 1: 29% *versus* 61%, *P *= 0.016 Cycle 2: 11% *versus* 47%, *P* = 0.003 CT Dose reduction in cycle 2: 11% *versus* 31% Better OM PRO with palifermin	Skin, mouth as above	84
Sarcoma (Phase II)	CT: six cycles, max; each 3 weeks doxorubicin: 90 mg/m^2^ ifosfamide: 10 g/m^2^ × 4 days or cisplatin: 120 mg/m^2^ (latter for osteosarcoma)	180 mcg/kg (single dose) 3 days pre-CT	Incidence, grade ≥ 3 OM: 13% *versus* 51%, *P *= 0.002 Incidence, grade ≥ 2 OM: 44% *versus* 88%, *P* < 0.001 Better PRO with palifermin	Skin, mouth as above, warm sensation, increased saliva	87

HDT-ASCT: high dose therapy-autologous stem cell transplant; CT: chemotherapy; RT: radiotherapy; TBI: total body irradiation; SRT: standard radiotherapy; HRT: hyperfractionated radiotherapy; 5-FU: fluorouracil; LV: leukovorin; OM: oral mucositis; PRO: patient-reported outcomes.

Results from palifermin cohort(s) are followed by results from the placebo group.

A post-marketing randomized clinical study raised a question regarding the efficacy of palifermin for mucositis prevention following CT-based conditioning regimens (Table 1) 35. Two hundred and eighty-one patients were randomized to receive palifermin or placebo in the setting of high-dose melphalan (1-day administration of 200 mg/m^2^ if creatinine clearance (CC) ≥30 ml/min. or 140 mg/m^2^ if CC <30 ml/min.) for multiple myeloma. Patients were assigned to receive palifermin either on a pre-ASCT schedule or a pre- and post-ASCT schedule. No difference in maximum severity of oral mucositis was seen. Severe oral mucositis (WHO grade 3 and 4) occurred in 37% (placebo), 38% (pre/post-ASCT) and 24% (pre-ASCT) (NS). There were more serious adverse events and total adverse events reported as treatment related in the arms that received palifermin compared with the placebo arm. The authors speculated that the lack of beneficial effect and increase in adverse events, particularly in the pre/post-arm, might have been a result of suboptimal timing of palifermin administration in this protocol. With the conditioning regimen completed in a single day, the palifermin post dose probably was given too early relative to the peak of oral mucositis, and the cumulative effects of six palifermin doses in close proximity to melphalan may have exacerbated the oral toxicity. A prior retrospective study had suggested that patients with normal renal function who had received only the three doses of palifermin before high-dose melphalan had a more benign hospital course, with a shorter hospital stay and reduced use of TPN and narcotics relative to historical controls 36. Following presentation of the post-marketing trial, the European drug regulatory authority limited the indication for palifermin to TBI-containing autologous transplants. Other recent trials, although smaller, have suggested that palifermin might have utility in regimens involving high-dose melphalan, specifically in ones where the risk for severe oral mucositis is greater. A phase I study of 19 patients with normal renal function receiving a melphalan-based autologous transplant for multiple myeloma evaluated escalation of melphalan dosing with palifermin administered pre and post transplant 37. In this trial, patients were able to tolerate an increase in the dose of melphalan to 280 mg/m^2^; escalation ended at that point because of cardiac toxicity. Two of six patients who received the highest melphalan dose did not suffer mucositis. Similarly, in another 19 patient study for patients with multiple myeloma and a CC ≤60 ml/min./1.73 m^2^, palifermin permitted a dose escalation of melphalan to 180 mg/m^2^ 38. Taken together with the positive results from the TBI-based ASCT trials 31–32, these findings suggest that palifermin is more likely to reduce oral mucositis when given with especially intensive conditioning regimens that would be expected to cause more mucosal damage.

### Oral mucositis following allogeneic transplantation

Published data on palifermin use following allo-HSCT are currently limited and no randomized placebo-controlled studies have been performed in this patient population. However, small prospective studies suggest that palifermin is also safe and prevents mucositis following allo-HSCT. Langner *et al*. 39 prospectively treated 30 patients who were receiving an allo-HSCT for leukaemia with palifermin using the approved pre/post-dosing schedule and compared these patients to a matched historical control group. They noted a decreased incidence in WHO grade 2–4 mucositis compared with control for patients who received palifermin (60% *versus* 80%, *P* = 0.04). They also noted a decrease in the mean duration of mucositis (6 *versus* 12 days, *P* = 0.003), a decrease in the mean number of days of TPN use (15 days *versus* 26 days, *P* = 0.002), and a decrease in the median cumulative dose of morphine equivalents given (150 mg *versus* 378 mg, *P* = 0.04) in patients who received palifermin. No apparent adverse effect of palifermin was seen in this study.

In another trial, Nasilowska-Adamska *et al*. 40 evaluated the ability of palifermin to prevent mucositis following both HDT-ASCT and allo-HSCT. A total of 53 patients with haematological malignancies were enrolled and received palifermin at the approved dosing. Twenty-four patients received an allogeneic transplant and the remainder of the patients received an autologous transplant. The results following palifermin administration were compared with a matched historical control group. The conditioning regimens included both TBI-based regimens and CT only-based regimens. All patients who received an allo-HSCT received GVHD prophylaxis that included methotrexate, which may impede palifermin's ability to prevent mucositis in allo-HSCT when palifermin is administered post stem cell infusion. Specifically, there is a concern that palifermin administered directly prior to methotrexate could induce epithelial cell entry into the cell cycle, rendering them more susceptible to the effects of methotrexate 41–42. However, palifermin appeared to be efficacious reducing the incidence, severity and duration of oral mucositis in a small study of patients with B cell malignancies who were treated with multiple cycles of high-dose methotrexate in a non-HSCT context 43. In the study by Nasilowska-Adamska *et al.,* a significant reduction in the incidence of WHO grade 1–4 (58% versus 94%, *P* < 0.001) and grade 3–4 mucositis (13% *versus* 43%, *P* < 0.001) was demonstrated with both the HDT-ASCT and allo-HSCT patients analysed together. Furthermore, the use of narcotic analgesics and TPN was also significantly reduced for the patients who received palifermin.

Based on pre-clinical studies that demonstrated a decrease in acute GVHD morbidity and mortality 44–47, Blazar *et al*. 48 performed a phase I/II dose-escalation, randomized, placebo-controlled trial that evaluated the effect of palifermin on the prevention of acute GVHD for patients receiving allo-HSCT. Sixty-nine patients received palifermin, while 31 patients received placebo. The study included four different dosing schedules of palifermin, with all patients receiving a minimum of three doses prior to the preparative regimen and three doses post stem cell infusion. The dosing schedules included: 40 mcg/kg/dose for six doses (total dose = 240 mcg/kg, *n* = 8), 60 mcg/kg/dose for six doses (total dose = 360 mcg/kg, *n* = 10), 60 mcg/kg/dose with an additional three doses post transplant (total dose = 540 mcg/kg, *n* = 14) and 60 mcg/kg/dose with an additional six doses post transplant (total dose = 720 mcg/kg, *n* = 37). Conditioning regimens included cyclophosphamide and TBI or cyclophosphamide and busulfan. Methotrexate with a calcineurin inhibitor was used for GVHD prophylaxis. A subgroup analysis of this study revealed a significant decrease in the incidence and mean severity of mucositis in patients who had received palifermin and been conditioned with a TBI-based preparative regimen, but not a CT-based preparative regimen. An analysis to look for a differential effect between dosing cohorts was not completed.

Finally, to further characterize palifermin's ability to mitigate mucositis following allo-HSCT, Goldberg *et al*. performed a retrospective study in 251 adult patients who received an allo-HSCT at Memorial Sloan-Kettering Cancer Center from 2004 to 2009 49. One hundred and fifty-four patients received the standard pre- and post-regimen of palifermin at 60 mcg/kg/dose (TBI-based = 77, CT based = 77) and 97 patients did not receive palifermin (TBI based = 44, CT based = 53). Data were collected on the number of days of patient controlled analgesia (PCA) use, TPN use and initial hospitalization for transplant as clinical surrogates for mucositis severity. Palifermin use in recipients of TBI-based allo-HSCT was significantly associated with fewer days on PCA (7 days *versus* 12 days, *P* = 0.033) and TPN (13 days *versus* 17 days, *P* < 0.001) and a decreased length of stay (32 days *versus* 38 days, *P* = 0.001). However, in recipients of a busulfan CT-based allo-HSCT, palifermin did not affect clinical surrogates of mucositis severity. Palifermin's ability to prevent mucositis in other CT-based preparative regimens was not assessed.

### Paediatric population

Preliminary results on 24 patients from a phase I dose-escalation study to evaluate the safety and pharmacokinetics of palifermin in paediatric patients receiving a myeloablative allo-HSCT have been described 50. Patients received three daily doses prior to a TBI-based preparative regimen and three daily doses post stem cell infusion. Three age groups were examined (1–2, 3–11, 12–16 years old). Three dose levels were studied including 40, 60 and 80 mcg/kg/dose. At the time of presentation, all cohorts were enrolled except for the 80 mcg/kg/dose for 1–2 year olds. No dose-limiting toxicities were seen in any age group. No grade 3 or 4 oral mucositis was seen in the 80 mcg/kg/dose cohorts. The data review team recommended a dose of 80 mcg/kg/dose for future efficacy studies in 3–11 and 12–16 year old age groups.

Another phase 1 dose-escalation study to evaluate the safety and pharmacokinetics of palifermin in paediatric patients receiving a myeloablative allo-HSCT was recently reported 51. Twelve children aged 2–18 were treated on the standard schedule (three daily doses prior to the conditioning regimen and three daily doses after stem cell infusion) at three doses (40, 60 and 90 mcg/kg/dose). Only three patients developed mucositis. All six patients who received palifermin at 90 mcg/kg/dose tolerated it without a dose-limiting toxicity. All patients experienced at least one adverse event, most of which were of NCI grade 1 or 2 in severity. The most predominant toxicity was a macular rash seen in eight patients. Linear kinetics was demonstrated for palifermin.

### Immune reconstitution

Several studies have indicated that immune recovery is an important predictor of survival following allogeneic transplant. For example, absolute lymphocyte count (ALC) has been demonstrated to be predictive of overall survival and relapse 52,53. Small *et al*. reported that the risk of opportunistic infections in the post-transplant period is correlated with the recovery of CD4 +  T cells 55. Goldberg *et al*. also showed that several measures of immune recovery, including ALC at day 30, natural killer cell count at day 60 and CD4 count at 6 and 12 months are important predictors of overall survival and disease-free survival following allogeneic transplant 56. Thus, promoting immune reconstitution following allogeneic transplantation is an important therapeutic goal.

Pre-clinical models have demonstrated that KGF plays an important role in T cell homeostasis and immune recovery through its role in regulating the proliferation and differentiation of thymic epithelium. Keratinocyte growth factor is necessary for thymic regeneration after radiation injury and administration of KGF prior to allo-HSCT resulted in an increase in thymopoiesis and peripheral T cell numbers post TBI-based allo-HSCT 57. Prior treatment with KGF enhanced responses to a DNA plasmid tumour vaccine after allo-HSCT, as indicated by increased numbers of tumour-specific CD8 +  T cells 58. Moreover, administration of KGF abrogated thymic changes caused by GVHD and preserved normal T lymphopoiesis in the setting of acute GVHD 59. Keratinocyte growth factor also promoted immune recovery following murine allogeneic umbilical cord blood transplant, as shown by an increased number of donor-derived T cells and NK cells in spleens of recipients who had received pre-transplant KGF 60. Improved thymic function was documented by a rise in the number of T cell receptor excision circles (TRECs) seen after KGF treatment. Other investigators have demonstrated that the post-HSCT recovery of T cells following KGF is further increased by sex hormone blockade using leuprolide 61. Furthermore, the effect of pre-HSCT KGF on recovery of thymic epithelial cells could be augmented by the administration of a p53 inhibitor during RT, resulting in more donor-derived CD4 +  and CD8 +  T cells 62. In the autologous HSCT setting, KGF stimulated thymus-mediated immune recovery in rhesus macaques as demonstrated by greater numbers of naïve T cells in lymph nodes and higher levels of TRECs following KGF administration 63.

Thus far, there have been no published prospective clinical trials evaluating palifermin for immune reconstitution. A retrospective study using the patient population included in the Blazar study 46 sought to evaluate if palifermin use in that trial affected ALC recovery 64. No relationship between palifermin use and ALC was demonstrated. However, there are now ongoing clinical trials designed to study this potential application of palifermin. At MSKCC, two separate phase II trials are currently enrolling patients to test palifermin's effects on immune reconstitution. One trial is a prospective single-arm study monitoring immune reconstitution following a CT-based allo-HSCT. In addition, MSKCC is enrolling patients into a prospective, randomized phase II trial comparing the effects on immune reconstitution of palifermin, palifermin with leuprolide acetate and placebo after TBI-based allo-HSCT. Patients are randomized in a 1:1:1 fashion. Also, the NIH has completed a trial studying the effectiveness of palifermin for promoting CD4 recovery in HIV patients (http://clinicaltrials.gov identifier NCT00376935), although the results are not yet available.

### Graft-versus-host disease

Considering its protective effect against epithelial tissue damage, most notably epithelial tissue in the gut, pre-clinical studies sought to assess if KGF was protective against GVHD 44–47. Several groups postulated that, based on the GI primacy model of acute GVHD 65–66, KGF administration would reduce GI tract damage and consequently result in less cytokine release, decreased gut bacteria translocation and, thus, a lower incidence of GVHD. These pre-clinical studies suggested that KGF may be a promising agent for the prevention of acute GVHD. In the first report, KGF administration on days 6 through 4 prior to BMT in mice ameliorated mortality, weight loss and GVHD-induced tissue damage in the liver, skin, lung and GI tract 45. In a follow-up study, the same investigators demonstrated that the mechanism of KGF's ability to prevent GVHD may be independent of repair of preparative regimen-associated injury 46. Moreover, KGF may improve engraftment. In another study, Krijanovski *et al*. 44 demonstrated in a murine model where GVHD is induced by both minor and major histocompatibility antigens that KGF administration from day −3 to +7 significantly reduced GVHD mortality and the severity of GI GVHD. Correlative studies demonstrated decreased serum lipopolysaccharide and tumour necrosis factor α levels. One possible mechanism for a decrease in GVHD in murine transplantation following KGF administration is that KGF may prevent or attenuate glutathione depletion and, thus, inhibit organ damage mediated by reactive oxygen species 47.

Building on these pre-clinical findings, clinical studies sought to evaluate palifermin's ability to protect against GVHD. Notably, as discussed above, Blazar *et al*. performed a randomized, placebo-controlled, phase I/II trial to answer this question 48. No difference in the rate of acute GVHD, time to engraftment, relapse or survival was noted between the two groups. Thus, although this study suggested that palifermin is safe to use in the context of allogeneic transplantation, it did not support the pre-clinical findings that palifermin may prevent acute GVHD. However, one potential limitation of this study was its varied dosing regimen as only 14 patients received palifermin at the currently approved dosing schedule 32. In addition, the use of methotrexate following palifermin administration may have abrogated the beneficial effect of palifermin through subsequent mucosal damage 41. A long-term follow-up of Blazar's study confirmed the original finding that palifermin did not prevent acute GVHD and further revealed that palifermin did not affect rates of chronic GVHD 67. Again, a survival benefit for palifermin was not demonstrated.

Nasilowska-Adamska *et al*. 40 also evaluated the effect of palifermin (60 mcg/kg/day for 3 days before and 3 days after conditioning regimen) on the rates of acute GVHD for the 24 patients in their study who had received an allo-HSCT. Among these patients, 71% had matched related donors and 29% had unrelated donors. Graft-versus-host disease prophylaxis consisted of cyclosporine and methotrexate for the related donors and cyclosporine, methotrexate and antithymocyte globulin for the unrelated donors. Compared with the historical control group, there was no difference in total grade of acute GVHD. However, when adding the total number of organs affected in each group, acute GVHD was less prevalent in the patients who received palifermin (*P* = 0.016). There was also a significant reduction in liver acute GVHD in patients who received palifermin (8.3% *versus* 37.5%, *P* = 0.036). A subsequent similar case-controlled study from the same group concluded that palifermin did not reduce the incidence of acute or chronic GVHD 68.

More recently, Jagasia *et al*. conducted a randomized, double-blind, placebo-controlled study to explore the potential of palifermin to reduce the incidence of severe acute GVHD in patients undergoing allo-HSCT for haematological malignancies from either a related donor or an HLA-matched, unrelated donor 69. Patients received placebo or palifermin 60 mcg/kg on three consecutive days before the conditioning regimen (six different options, including ones with CT alone and others that combined CT with TBI) and a single dose of 180 mcg/kg after conditioning, but often 1–2 days before allo-HSCT. Patients also received methotrexate plus cyclosporine or tacrolimus on days 1, 3, 6 and 11 for GVHD prophylaxis. The incidence of grade 3 or 4 acute GVHD (17% placebo and 16% palifermin), grade 3 or 4 oral mucositis (73% placebo and 81% palifermin) were similar between treatment groups. The most common treatment-related adverse events were skin rash, pruritus and erythema. In this study, using this dosing regimen of palifermin, there was no positive impact on the incidence or severity of acute GVHD or mucositis.

In summary, thus far, multiple small clinical trials have not borne out the observations in mouse models that palifermin can inhibit GVHD. Larger, controlled prospective studies are needed to more definitively determine if palifermin can be efficacious in this setting. Such efforts should take into account different conditioning regimens (with or without TBI), GVHD prophylaxis (with or without methotrexate) and attempt to optimize the dosing schedule of palifermin for this indication. More prolonged administration of palifermin may be necessary to limit acute and chronic GVHD.

## Concurrent chemotherapy and radiation for solid tumours

### Oral mucositis in head and neck cancer

Fractionated RT administered concomitantly with CT is the standard treatment for patients with locally advanced head and neck cancer not undergoing resection. Oral mucositis is an almost universal complication of combined CT/RT and presents a major clinical and economic burden in these patients. Resulting severe pain and difficulty in swallowing can be debilitating and affect their nutritional status leading to dehydration, weight loss and treatment interruption, and can adversely influence treatment outcome. Given the unmet need for effective therapy for oral mucositis, a number of studies have investigated the role of palifermin in this setting.

In a multicenter, double-blind, randomized, phase II study 70, patients with advanced head and neck cancer receiving concurrent CT/RT were randomized to palifermin 60 mcg/kg (67 patients) or placebo (32 patients) once weekly for 10 doses (Table 1). Standard RT (daily 2-Gy fractions to 70 Gy) or hyperfractionated RT (1.25-Gy fractions twice daily to 72 Gy) was delivered over 7 weeks. Concurrent CT consisted of cisplatin 20 mg/m^2^/day and 5-FU 1000 mg/m^2^/day for 4 days. In this study, palifermin administered at a dose of 60 mcg/kg weekly during CT/RT appeared to reduce mucositis, dysphagia and xerostomia with hyperfractionated RT but not with standard RT. However, the positive results with hyperfractionated RT were not statistically significant and the authors concluded that higher doses of palifermin should be used in future studies. Subsequently, two double-blind randomized, placebo-controlled phase III trials were conducted in this patient population.

The first study involved 186 patients with locally advanced head and neck cancer who had a partial or complete resection of their tumour prior to CT/RT (Table 1) 71. The post-operative treatment included RT, 60–66 Gy (2 Gy per fraction and five fractions per week) and concurrent CT with cisplatin 100 mg/m^2^ on days 1 and 22. Palifermin at a weekly dose of 120 mcg/kg or placebo was administered 3 days before and throughout CT/RT. In this study, palifermin reduced the incidence of oral mucositis to 51% as compared with 67% with placebo (*P* = 0.027), and mean duration of mucositis was reduced from 22 days to 4.5 days. Most adverse events related to palifermin were mild and severe adverse events were typical of CT/RT. Grades 3 or 4 increases in amylase levels were seen more often in the palifermin arm than in the placebo arm (50% *versus* 42%). The amylase levels returned to normal by week 3. Anti-palifermin antibodies developed in some patients on both treatment arms (seven palifermin and five placebo), but were non-neutralizing. The overall survival (HR, 0.96, 95% CI 0.54–1.71) and progression-free survival (HR, 1.01, 95% CI 0.60–1.69) were similar in the two treatment arms (median follow-up 32.8 months).

In the second study, patients with locally advanced head and neck cancer but no prior surgery received palifermin at a dose of 180 mcg/kg (*n* = 94) or placebo (*n* = 94) before starting CT/RT and then once weekly for 7 weeks (Table 1) 72. In this study, palifermin reduced the incidence of severe oral mucositis from 69% to 54% (*P* = 0.041), the mean duration of severe oral mucositis from 26 to 5 days, and delayed the median time to onset of severe oral mucositis. However, the use of opioid analgesics, mouth and throat soreness scores, and CT/RT compliance were not significantly different between treatment arms, possibly related to the sample size. The most common adverse events included skin rash, flushing and dysgeusia. The progression-free survival and overall survival were similar in both treatment groups.

In both studies, palifermin significantly reduced the incidence of severe oral mucositis and there was no statistically significant difference between palifermin and placebo for the progression-free survival or overall survival. However, palifermin did not significantly alter mouth throat soreness (MTS) scores in either of these studies. The higher baseline MTS score in these studies compared with the haem transplant trials presumably was related to the tumour or post-surgical pain, and may not be affected by the palifermin treatment. Nonetheless, the lack of a beneficial effect based on patient-reported outcome and the modest improvement in other criteria indicated that additional optimization of the palifermin dose/schedule is necessary to demonstrate that palifermin would be safe and effective in limiting the impact of oral mucositis in patients subjected to toxic head/neck cancer treatment regimens.

### Dysphagia in lung cancer

Concurrent CT/RT (CT/RT) is the standard treatment for locally advanced and unresectable non-small cell lung cancer (NSCLC). However, it is characterized by a high risk of oesophagitis and dysphagia, as well as other treatment-related toxicities 73–76. Oesophagitis and dysphagia may be severe and disabling, resulting in pain, weight loss, hospitalization and the need for a gastrostomy or jejunostomy tube for enteral feeding. RT and CT interruptions may be necessary to allow for recovery of the oesophageal lining, although decreases in CT/RT dose intensity can have an adverse impact on tumour control and survival 77,78.

In a randomized phase II trial involving adult patients with unresectable stage III NSCLC, Schuette *et al*. assessed the efficacy and safety of palifermin in reducing dysphagia from CT/RT followed by consolidation CT (Table 1) 80. Patients received weekly paclitaxel (50 mg/m^2^) and carboplatin (AUC 2.0) with concurrent daily RT to a total of 60–66 Gy, followed by two cycles of consolidation CT with paclitaxel (225 mg/m^2^) and carboplatin (AUC 6.0). Palifermin 180 μg/kg (*n* = 49) or placebo (*n* = 46) was administered 3 days before starting concurrent CT/RT, and then once weekly for 6 weeks (total of seven doses). The primary end-point of the study was incidence of grade ≥2 dysphagia evaluated using the Common Terminology Criteria for Adverse Events, Version 3.0 (CTCAE v3.0) dysphagia scale. Secondary end-points included additional dysphagia measures, change in Eastern Cooperative Oncology Group (ECOG) performance status and incidence of unplanned breaks or discontinuations of RT. Safety end-points included adverse events as well as tumour response rate, progression-free survival and overall survival.

Results demonstrated a numerically lower incidence of grade ≥2 dysphagia in the palifermin group (61%) than in the placebo group (70%, *P* = 0.36). Lower values were also reported in the palifermin group than in the placebo group for the incidence of grade ≥3 dysphagia (22% *versus* 28%, *P* = 0.50), the mean duration of grade ≥2 dysphagia (25.3 days *versus* 32.4 days, *P* = 0.32) and the incidence of unplanned RT breaks (18% *versus* 33%, *P* = 0.11). Median time to onset of grade ≥2 dysphagia was 45 days in the palifermin group and 31 days in the placebo group (*P* = 0.21).

Patients in the palifermin group received more doses of study drug (palifermin) than patients in the placebo group: 42 patients (86%) in the palifermin group and 30 (65%) in the placebo group received 7 or 8 doses. The mean (SD) total dose of RT for patients who received palifermin was 58.3 (8.4) Gy and 52.2 (16.1) Gy for the placebo group. The number of patients receiving a cumulative RT dose ≥60 Gy was 41 (84%) in the palifermin group and 28 (61%) in the placebo group (*P* = 0.01). Similarly, patients in the palifermin group received more doses of paclitaxel and carboplatin than patients in the placebo group: 35 patients (71%) in the palifermin group and 28 (61%) in the placebo group received six or more doses. The mean (SD) relative dose intensity for carboplatin was 93% (22%) for the palifermin group and 74% (27%) for the placebo group, and for paclitaxel was 90% (15%) for the palifermin group and 81% (26%) for the placebo group.

The incidence of adverse events was similar in the two treatment groups. Serious adverse events were reported for 44% of patients in the palifermin group and 65% in the placebo group. Fatal adverse events were reported for seven patients, two (4%) in the palifermin group and five (11%) in the placebo group; none of these adverse events was considered by the investigator to be related to palifermin treatment. Median overall survival and progression-free survival were not adversely affected by palifermin treatment, with median time to death of 513 days for the palifermin group and 319 days for the placebo group [*P* = 0.42; hazard ratio (HR) 0.8, 95% CI 0.4–1.5]. Median time to disease progression or death was 262 days for the palifermin group and 235 days for the placebo group (*P* = 0.20; HR 0.7, CI 0.4–1.2). Overall tumour response rate was numerically better for patients in the palifermin group; 33 of 48 patients (69%) in the palifermin group and 22 of 46 (48%) in the placebo group had a complete or partial response. In the long-term safety evaluation at month 6, patients in the placebo group had a 26% incidence of tumour progression or recurrence, as compared with 19% in the palifermin group.

This was a first, hypothesis-generating study to assess the effect of palifermin on dysphagia in patients with unresectable stage III NSCLC receiving concurrent CT/RT. Although the study was exploratory and had a small sample size, the results suggest a possible benefit from palifermin in reducing the incidence, duration and time to onset of dysphagia, and in increasing the RT and CT dose applied without negatively affecting survival. Greater exposure to CT/RT in the palifermin group apparently was because of a higher treatment discontinuation rate in the placebo group, resulting in patients who received fewer of the scheduled CT/RT doses. Increased exposure to CT and RT may have contributed to higher response rates in the palifermin arm and the numerical difference in survival, as several studies have indicated a relationship between higher doses of CT and RT and local tumour control in NSCLC. 81,82 No major acute or chronic safety concerns were identified for palifermin. The authors concluded that additional, larger studies are warranted to further assess the potential benefit of palifermin with CT/RT in NSCLC.

## Chemotherapy with mucotoxic agents for solid tumours

Mucositis can be a significant toxicity in patients treated with several chemotherapeutic agents such as 5-FU or doxorubicin administered by continuous infusion. The rapidly proliferating epithelial cells of the oral and GI tract are very sensitive to cytotoxic agents, especially cell cycle-specific agents, resulting in oral, oesophageal and/or intestinal mucositis, which can be dose-limiting.

### Colorectal cancer

The initial safety profile and the maximally tolerated dose for palifermin were determined in a phase I clinical trial of patients with metastatic colorectal cancer. 29. 5-FU and leucovorin (LV)-based regimens are commonly used in the treatment of colorectal cancer. Gastrointestinal mucositis and diarrhoea can be dose-limiting toxicities of 5-FU/LV, resulting in dose reduction and treatment delays. As noted above, the study of patients treated with 5-FU indicated a trend towards a reduced frequency of grade 2–4 oral mucositis with palifermin (43% *versus* 67%, *P* = 0.06) and a maximum tolerated dose of 80 mcg/kg/day, administered for three consecutive days before one cycle of CT 29.

In part two of the study, Rosen *et al*. investigated the efficacy of palifermin (*n* = 28) *versus* placebo (*n* = 36) in reducing mucositis and diarrhoea when administered at a dose of 40 mcg/kg for 3 days prior to two CT cycles (Table 1) 84. Although no statistically significant differences were observed in the incidence and severity of diarrhoea, the incidence of WHO grade 2 or higher oral mucositis was significantly lower (29% *versus* 61% in cycle 1 and 11% *versus* 47% in cycle 2) and dose reduction in cycle 2 was less frequent in the palifermin group as compared with the placebo group (14% *versus* 31%). Treatment with palifermin was associated with mild-to-moderate oral-related toxicities such as white coating of the tongue and taste disorders, skin-related adverse events such as erythema and pruritus, and transient increases in serum amylase and lipase levels.

### Sarcoma

Doxorubicin is a very effective agent in the treatment of soft tissue sarcoma, and is often combined with ifosfamide or dacarbazine (AI or ADIC regimens). The incidence of significant oral mucositis (grade 2 or higher) with doxorubicin administered by continuous intravenous infusion is more than 75%, with nearly half the patients experiencing severe mucositis 85–86. The management of patients experiencing severe mucositis involves treatment delays until recovery of mucosal tissues, dose reduction in CT and/or reduction in the infusion time of doxorubicin from 3 to 2 days or administration by bolus infusion. Dexrazoxane is often used for cardioprotection when doxorubicin is administered by bolus infusion, as cardiac toxicity may be increased. Because reduction in the dose and treatment delays may compromise treatment outcome, it is important to develop novel strategies to prevent and alleviate severe mucositis in this setting.

Based on these observations, Vadhan-Raj and colleagues investigated palifermin in patients with soft tissue sarcoma undergoing multiple cycles of intensive CT with doxorubicin administered at 90 mg/m^2^ by continuous intravenous infusion over 72 hrs along with ifosfamide 10 g/m^2^ (AI regimen) or cisplatin 120 mg/m^2^ (AP regimen) (Table 1) 87. In this double-blind, placebo-controlled study, patients were randomized 2:1 to receive a single intravenous dose of palifermin 180 mcg/kg (*n* = 32) or placebo (*n* = 16) 3 days prior to each CT cycle for up to a maximum of six cycles. Patients experiencing severe mucositis (WHO grades 3 or 4 mucositis) were allowed to receive open-label palifermin (180 mcg/kg) for the subsequent cycles. The rationale for choosing a single dose of 180 mcg/kg, instead of three daily doses of 60 mcg/kg before the regimen as approved in the transplant setting, was two-fold. Firstly, administration of multiple doses of palifermin up to the day before CT may sensitize the rapidly proliferating mucosal tissue to CT-induced injury. Secondly, administration of a single dose 3 days before CT would be more convenient for patients.

In this study, palifermin significantly reduced the incidence, severity and duration of mucositis as compared with placebo. The incidence of grade 2 or higher mucositis was reduced by half (from 88% to 44%, *P* < 0.001) and grades 3 or 4 mucositis by three quarters (from 51% to 13%, *P* = 0.002). The duration of severe mucositis was also reduced by half (from 6 to 3 days). As a result, 63% of patients receiving palifermin completed six blinded cycles as compared with only 31% of patients receiving placebo. Furthermore, all seven patients from the placebo group who had experienced severe mucositis and subsequently crossed over to open-label palifermin avoided severe mucositis. Thus, palifermin when used as a secondary prophylaxis was effective in preventing the recurrence of severe mucositis in high-risk patients with prior mucosal injury. The reduction in mucositis in these patients translated into clinical benefits, including amelioration of mouth and throat soreness, reduction in the need for opioid analgesics and improvement in ability to drink, eat and talk. The palifermin group also experienced less severe nausea, possibly related to less mucosal damage to the alimentary tract. The tumour response and progression-free survival were not different in the two arms.

Results from the objective oral assessment correlated very well with the subjective assessment of the patients in their daily symptom record diaries (weighted k, 0.63, *P* < 0.001). In addition, the median scores of mouth, throat and rectal soreness were significantly lower and scores for daily living activities significantly better in the palifermin group as compared with the control group.

## Biological response to palifermin in tissue samples

Palifermin exhibits many biological activities that may contribute to mucosal protection 15. In the study by Vadhan-Raj *et al*., mucosal biopsy samples were examined before and after the first dose of palifermin to better understand the nature of the tissue response 87. Buccal biopsies showed epithelial hyperplasia with a marked increase in the proliferative marker, Ki67 in five of seven patients after palifermin treatment. These findings suggested that mucosal hypertrophy was one of the mechanisms of mucosal protection, increasing the tissue reservoir prior to CT. Consistent with these observations, many patients perceived an increase in the thickness of their tongue, as had been noted in other clinical studies. One potential concern is that the rapidly proliferating mucosal cells could be sensitized to CT. However, the increased expression of cyclin E, a putative G1 marker, suggested that most of the mucosal cells were not in S phase just before CT and therefore not predisposed to damage from the chemotherapeutic agents.

## Adverse effects and potential concerns

As described above, palifermin has been generally well tolerated, with the typical side effects being mild to moderate in severity, consisting of temporary alterations in taste, apparent thickening of the tongue and buccal mucosa, white coating of the tongue, erythema and burning sensation in the skin, rash, pruritus and transient elevation in blood levels of amylase and lipase. There have been some case reports describing more serious cutaneous toxicity involving palmar-plantar erythrodysaesthesia, which might increase susceptibility to CT-induced toxicity, as described with cytarabine 88–89.

Because palifermin is a mitogen for epithelial cells and many epithelial tumours express FGFR2b, there are potential concerns that palifermin might promote tumourigenesis by stimulating tumour cell growth, inhibiting apoptosis or protecting the cells from cancer therapy. Thus far, the clinical studies of palifermin in patients with solid tumours or haematological malignancies have not shown signs of such adverse effects. However, long-term follow-up of patients treated with palifermin in clinical trials is ongoing to ensure its safety.

## Summary and future directions

Mucositis is a highly feared and potentially debilitating toxicity associated with many cancer therapies. It has a significant impact on patients' quality of life, morbidity and treatment outcome, as well as health care cost. Based on pre-clinical data, clinical studies demonstrated that palifermin mitigates oral mucositis in patients after CT/RT and autologous HSCT. Specifically, in a randomized, placebo-controlled phase III study, palifermin decreased the incidence and severity of oral mucositis following TBI-based HDT-ASCT. Thus far, limited data suggest that this effect will translate to patients receiving allo-HSCT. Currently, palifermin has not been shown to ameliorate oral mucositis in patients treated with CT-based autologous or allo-HSCT. Additional trials with different CT regimens and perhaps variation in the dose/schedule of palifermin would provide valuable information about its use in these settings.

Pre-clinical data have suggested that palifermin might promote immune recovery following allo-HSCT. Ongoing studies are assessing if these findings will be confirmed clinically. Pre-clinical data also indicated that palifermin might prevent GVHD following allo-HSCT. Presently, there is no clinical evidence that palifermin can prevent GVHD following allo-HSCT. Larger prospective studies that test different palifermin dosing regimens as well as various conditioning and immunosuppressive regimens may help answer this question.

Although beneficial effects of palifermin also have been observed in patients with solid tumours, currently regulatory approval has not been extended to these settings. Two double-blind, placebo-controlled phase III trials showed that a weekly dose of palifermin was safe and effective in reducing the incidence of severe oral mucositis in patients with head and neck cancer who were treated for several weeks with CT/RT. However, the magnitude of palifermin's protective effect was relatively small and was not reinforced by the patients' self-assessment. Further studies are needed to obtain definitive information about its use in this context. Similarly, additional trials are required to determine the utility of palifermin in patients with NSCLC undergoing CT/RT, where a reduction in dysphagia would be a primary end-point. Considering the distribution of the KGF receptor and biological effects observed in pre-clinical studies, it is possible that palifermin would alleviate lower GI mucositis in patients receiving various forms of CT/RT, especially ones including pelvic RT.

Encouraging results were obtained when palifermin was given to patients with sarcoma who were being treated with mucotoxic CT. Because these tumours lack expression of the KGF receptor, the theoretical possibility that palifermin would have direct effects on the tumour cells that could limit the success of cancer therapy is low. Of particular note, in this study, patients in the placebo arm, who had experienced severe oral mucositis following their initial cycles of CT, were almost uniformly spared this severe side effect when they were given open-label palifermin 87. This implied that selective use of palifermin in patients who had suffered from severe mucositis in early rounds of chemo/radiotherapy would be a cost-effective approach to minimize discomfort and enable safe delivery of chemo/radiotherapy in the subsequent rounds of therapy. Additional larger studies with a crossover design should be performed to test this idea. Future work with palifermin should also evaluate alternatives to the schedule and dose approved in the TBI-based haem transplant setting. Clinical experience has indicated that variations in the timing and magnitude of palifermin doses are often well tolerated and efficacious. A flexible approach to palifermin dosing should optimize its beneficial effects in trials involving different cancer treatment regimens.
